# Inflammatory factors and nasopharyngeal carcinoma: A two-sample Mendelian randomization analysis

**DOI:** 10.1097/MD.0000000000044400

**Published:** 2025-09-26

**Authors:** Keyun Lin, Jiaxiang Hu, Guanying Qiao, Mei Jiang

**Affiliations:** a Department of Oncology, First Clinical Medical College, Guangzhou University of Chinese Medicine, Guangzhou, Guangdong Province, China; b Department of Oncology I, ShunDe Hospital Guangzhou University of Chinese Medicine, Foshan, Guangdong Province, China; c Department of Oncology, First Affiliated Hospital, Guangzhou University of Chinese Medicine, Guangzhou, Guangdong Province, China.

**Keywords:** granulocyte colony-stimulating factor, inflammatory factors, Mendelian randomization, nasopharyngeal carcinoma, tumor microenvironment

## Abstract

Nasopharyngeal carcinoma (NPC) is an important health concern worldwide. Previous studies are susceptible to confounding factors. To solve this problem, this study uses Mendelian randomization (MR) to discover the causal relationship between inflammatory factors and NPC from a genetic perspective. A 2-sample MR analysis was performed using data from genome-wide association analysis studies of 41 inflammatory factors and NPC. The following methods were used to analyze the causal relationship between inflammatory factors and NPC: Inverse-Variance Weighted, MR-Egger, Weighted Median, Simple Mode and Weighted Median. MR Steiger test was used to determine the direction of the interaction between inflammatory factors and NPC. The robustness of the analysis was ensured by means of Cochran Q test, leave-one-out analysis and MR-Egger regression analysis. Reverse MR was performed to investigate whether there is reverse causality between inflammatory factors and NPC. There was a positive causal relationship between granulocyte colony-stimulating factor (G-CSF) levels and NPC (*OR*: 3.659, 95% *CI*: 1.398–9.581, *P* = .008), and there was no pleiotropy or reverse causality between the level of G-CSF and NPC. The sensitivity analysis showed that the results of the analyses did not contain heterogeneity, horizontal pleiotropy, or individual single nucleotide polymorphisms that significantly influenced the results of the analyses. G-CSF is a potential risk factor for NPC. The results of this study may provide new research ideas for identifying tumor markers and therapeutic targets for NPC.

## 1. Introduction

Nasopharyngeal carcinoma (NPC) is a malignant tumor originating from the nasopharyngeal epithelium with obvious epidemiological characteristics of regional aggregation and population susceptibility. The Chinese population exhibits a high incidence of NPC, with the Guangdong province of China demonstrating a particularly elevated rate of this malignancy. This observation has led to the colloquial designation of the disease as “Guangdong carcinoma.”

NPC demonstrates highly sensitive to radiotherapy and chemotherapy. Patients often experience enhanced therapeutic outcomes following systematic treatment regimens. However, these treatments are associated with numerous adverse reactions, which can result in physiological and psychological dysfunction. Consequently, patients may experience a decline in their quality of life and a decrease in treatment motivation. NPC is characterized by its high degree of invasiveness, which is manifested in its biological behavior. This invasive nature leads to strong regional invasion and distant metastasis, resulting in a high risk of recurrence and metastasis after treatment. Local recurrence and distant metastasis are the primary factors contributing to mortality in cases of NPC.^[1,2]^ Therefore, it is imperative to identify a treatment that is both efficacious and devoid of deleterious side effects, while also ensuring its sustainability to enhance the quality of life and extend the survival duration of patients.

The tumor microenvironment (TME) is composed of tumor cells and a multitude of other cell types. It is the milieu that fosters tumorigenesis and tumor development. Tumor cells secrete an array of inflammatory factors and recruit inflammatory cells, which form an integral component of the tumor microenvironment. Tumor-related inflammation is a hallmark manifestation of tumors. Inflammation pathways activated by inflammatory factors, such as PI3K/Akt, MAPK/ERK, NF-κB and JAK/STAT, etc, are classic tumor-related pathways. The tumor microenvironment establishes an internal milieu conducive to tumor growth, metastasis, immune escape, and angiogenesis by mediating common pathways of inflammation and tumors. Anti-inflammatory therapy is regarded as a means of mitigating the occurrence of tumors and the associated risk of mortality, signifying a prospective avenue for antitumor treatment.

NPC has been shown to be associated with Epstein–Barr virus infection. Epstein–Barr virus has been shown to induce an inflammatory response in NPC cells, thereby promoting the production of inflammatory factors and contributing to the formation of the TME of NPC.^[3,4]^ In addition, the inflammatory microenvironment, under the long-term influence of inflammatory factors such as CCL3, CCL4, IL-1β, IL-6, IL-8 and IL-10, increases the risk of NPC pathogenesis and metastasis.^[5]^ The present study was guided by the hypothesis that inflammatory responses have a carcinogenic effect on malignant tumors.

To investigate the causal relationship between inflammatory factors and the incidence of NPC, genetic instrumental variables (IVs) were derived through Mendelian randomization (MR). We hope that our study will provide ideas and references for future research on the prevention and treatment of NPC.

## 2. Materials and methods

### 2.1. Study design

The causal relationship between 41 inflammatory factors and NPC is to be evaluated based on a 2-sample MR analysis. 41 inflammatory factors were utilized as exposure factors, NPC as an outcome factor, and genetic variants with significant statistical significance in the inflammatory factors, namely single nucleotide polymorphisms (SNPs), were selected as IVs for the 2-sample MR analysis.

### 2.2. Data sources

A publicly available genome-wide association study (GWAS) database was selected for the present analysis, and the data on exposure factors and outcome factors were obtained from the Finnish population in Europe. Refer to previous studies, the data for 41 inflammatory factors were obtained from an investigation included in the NHGRI-EBI Catalog database (https://www.ebi.ac.uk/gwas) concerning the genetic associations of circulating inflammatory factors in 8293 Finns (GWAS ID: ebi-a-GCST004458).^[6,7]^ This investigation encompasses population cohort data from 3 autonomous studies: the “Cardiovascular Risk in Young Finns study,” “FINRISK1997,” and “FINRISK2002.” Detailed information on these 41 inflammatory factors can be found in Supplementary Material, Supplemental Digital Content, https://links.lww.com/MD/Q57. The data for NPC were derived from a research project entitled “Malignant neoplasm of nasopharynx (controls excluding all cancers)” in the FINNGEN database(https://r11.finngen.fi). The data for NPC were based on the results of 89 cases of NPC compared with 345,118 healthy individuals. A detailed overview of the GWAS data in this study are presented in Table 1.

**Table 1:** Information on the datasets for inflammatory factors and NPC.

Trait	Population	Year	Sample size	Number of SNPS
Interleukin-1 receptor antagonist	European	2016	3638	9564,741
Interleukin-1-beta	European	2016	3309	9983,642
Interleukin-2	European	2016	3475	9512,914
Interleukin-2 receptor,alphasubunit	European	2016	3677	9583,519
Interleukin-4	European	2016	8124	9786,064
Interleukin-5	European	2016	3364	9450,731
Interleukin-6	European	2016	8189	9790,590
Interleukin-7	European	2016	3409	9692,306
Interleukin-8 (CXCL8)	European	2016	3526	9517,348
Interleukin-9	European	2016	3634	9567,876
Interleukin-10	European	2016	7681	9793,415
Interleukin-12p70	European	2016	8270	9799,886
Interleukin-13	European	2016	3557	9539,073
Interleukin-16	European	2016	3483	9551,485
Interleukin-17	European	2016	7760	9786,653
Interleukin-18	European	2016	3636	9785,222
Tumor necrosis factor-alpha	European	2016	3454	9500,449
Tumor necrosis factor-bata	European	2016	1559	6304,298
TNF-related apoptosis inducing ligand	European	2016	8186	9698,525
Interferon-gamma	European	2016	7701	9785,363
Macrophage migration inhibitory	European	2016	3494	9537,573
Stem cell factor	European	2016	8290	9796,683
Macrophage colony-stimulating factor	European	2016	840	9184,521
Granulocyte colony-stimulating factor	European	2016	7904	9788,961
Beta nerve growth factor	European	2016	3531	9537,863
Hepatocyte growth factor	European	2016	8292	9802,538
Basic fibroblast growth factor	European	2016	7565	9790,946
Platelet derived growth factor BB	European	2016	8293	9800,009
Vascular endothelial growth factor	European	2016	7118	9784,803
Stem cell growth factor beta	European	2016	3682	9574,890
Monocyte chemotactic protein-1 (CCL2)	European	2016	8293	9801,908
Macrophage inflammatory protein-1α(CCL3)	European	2016	3522	9519,267
Macrophage inflammatory protein-1β(CCL4)	European	2016	8243	9802,973
Regulated on activation, normal T-cell expressed and secreted (CCL5)	European	2016	3421	9523,827
Monocyte specific chemokine 3 (CCL7)	European	2016	843	7630,881
Eotaxin(CCL11)	European	2016	8153	9793,404
Cutaneous T-cell attracting (CCL27)	European	2016	3631	9568,408
Growth regulated oncogene-α(CXCL1)	European	2016	3505	9528,505
Monokine induced by interferon-gamma (CXCL9)	European	2016	3685	9579,894
Interferon-gamma-induced protein 10 (CXCL10)	European	2016	3685	9576,881
Stromal cell-derived factor-1 alpha (CXCL12)	European	2016	5998	9736,366
Nasopharyngeal carcinoma	European	2023	345,207	20,442,898

NPC = Nasopharyngeal carcinoma.

### 2.3. IVs selection

**Figure 1.**
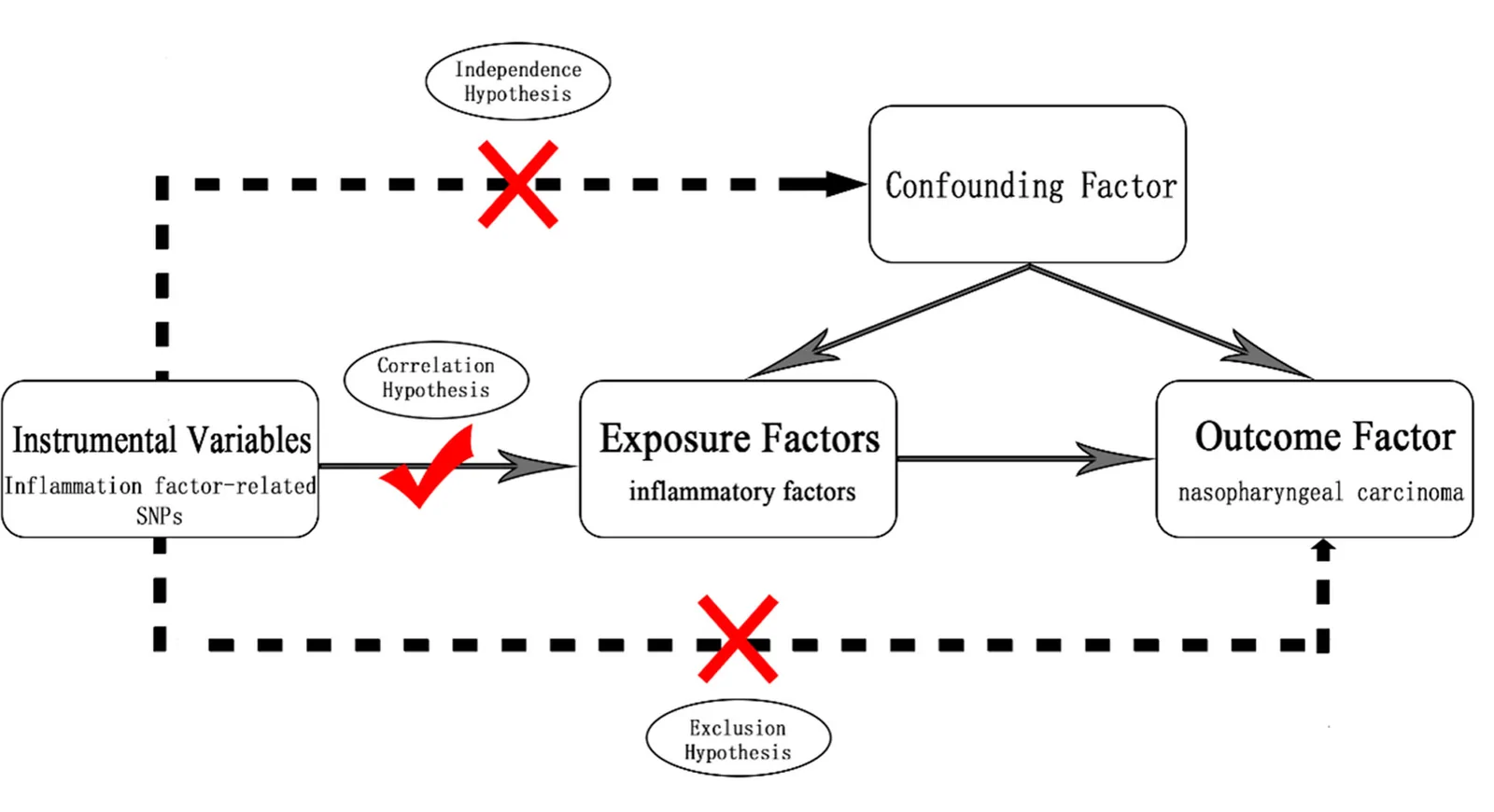
Schematic of MR analysis assumptions. MR = Mendelian randomization.

The IVs in this study met the following hypotheses (Fig. 1): correlation hypothesis: IVs are strongly associated with exposure factors; independence hypothesis: IVs are not associated with confounders; exclusion hypothesis: IVs influence outcome factors only through exposure factors. In selection process of IVs, given that there were few SNPs satisfying the *P* < 5 × 10^−8^ condition, the SNPs significance level was set to *P* < 5 × 10^−6^ to ensure relevance^[8]^; for linkage disequilibrium SNP elimination, set the coefficient to *r*^*2*^ < 0.001 and kb < 10000; based on the formula $F=\frac{R^{2}\left(n-1-k\right)}{\left(1-R^{2}\right)k}$ and $R^{2}=2\times \left(1-MAF\right)\times MAF\times \frac{\beta }{SD}$ to remove SNPs with *F* ≤ 10 to avoid weak IVs correlation bias^[9]^; remove SNPs associated with confounders using the LDlink database (https://ldlink.nih.gov/), remove palindromic sequences to achieve allelic alignment and eliminate duplicate SNPs. In the reverse MR analysis, the SNP screening criteria we applied was the same as the forwards MR analysis, which is *P* < 5 × 10^−6^.

### 2.4. Statistical analysis

Inverse-Variance Weighted (IVW), MR-Egger, Weighted Median, Simple Mode, and Weighted Mode were selected for MR analysis. The IVW method has the highest test efficiency when horizontal pleiotropy of IVs does not exist; therefore, it was used as the primary analysis method.^[10]^ The Bonferroni correction formula is 0.05/ (number of exposures included in the study × number of outcomes included in the study).^[11]^ Therefore, in the study, *P*-values of <.0012 (0.05/41) were considered to indicate a strong association, and *P*-values between .0012 and .05 were considered to indicate a suggestive association.

To ensure the robustness of the analysis results, Cochran *Q* test was used to assess heterogeneity in IVW and MR-Egger; the leave-one-out analysis was used to test whether a single SNP had an excessive influence on the results of the MR analysis, and MR-Egger regression analysis was used to test for horizontal pleiotropy and visualize the analysis results.

In the reverse MR analysis, genetic data of NPC were used as exposure, while genetic data of 41 inflammatory factors were used as outcome to determine whether there was a reverse causality between NPC and inflammatory factors. The screening criteria for SNPs were as serious as those in the forwards MR analysis.

The Steiger direction test is a methodological instrument employed for the purpose of analyzing to analyze and subsequently determining the direction of the causal relationship between exposure and outcome factors. The utilization of Steiger direction test guarantees a robust correlation between the exposure factor and SNPs, while ensuring that the SNPs exclusively influence the outcome factor through the exposure factor. This methodological framework precludes the occurrence of bias owing to reverse causality.^[12]^

### 2.5. Statistical analysis software settings

Statistical analysis was conducted using the “TwoSampleMR” package in R software (version 4.4.1),^[13,14]^ with a statistical significance threshold of *P* < .05. The relationship between exposure and the outcome factors was quantified using odds ratio (OR). An OR of 1 indicated that the exposure factor was not associated with the outcome factor. An OR >1 indicates that the exposure factor is a risk factor for the outcome factor, while an OR <1 indicates that the exposure factor is a protective factor for the outcome factor.

## 3. Results

### 3.1. IVs

Following the elimination of linkage disequilibrium, SNPs associated with confounding factors, SNPs with reverse sequences, and SNPs with weak IVs. For example, a total of 8 SNPs remained for G-CSF. The number of SNPs that meet the requirements for all 41 inflammatory factors can be ascertained in the additional file.

### 3.2. The forwards MR analysis

To account for any linkage disequilibrium, we excluded IVs with an *r*^2^ value below 0.01 within a range of 1000KB. The calculation formula of the F value was used to value the *F* values of the IVs selected in this study. The effect of them were found to be strong, because the *F* values were all >10. No confounding factors were association with NPC.

**Figure 2.**
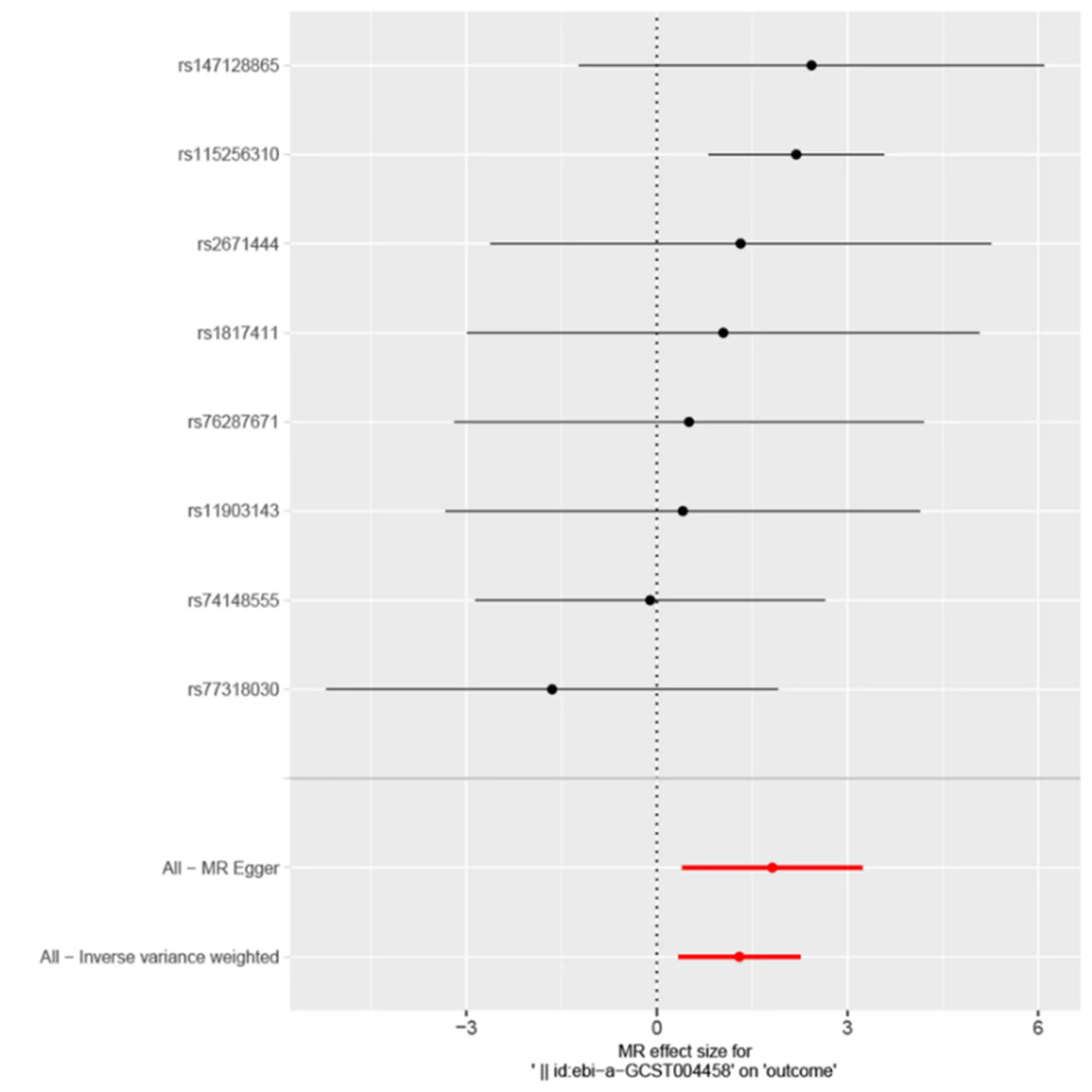
Forest plot of MR analysis of G-CSF levels and NPC. C-GSF = granulocyte colony-stimulating factor, MR = Mendelian randomization, NPC = nasopharyngeal carcinoma.

**Figure 3.**
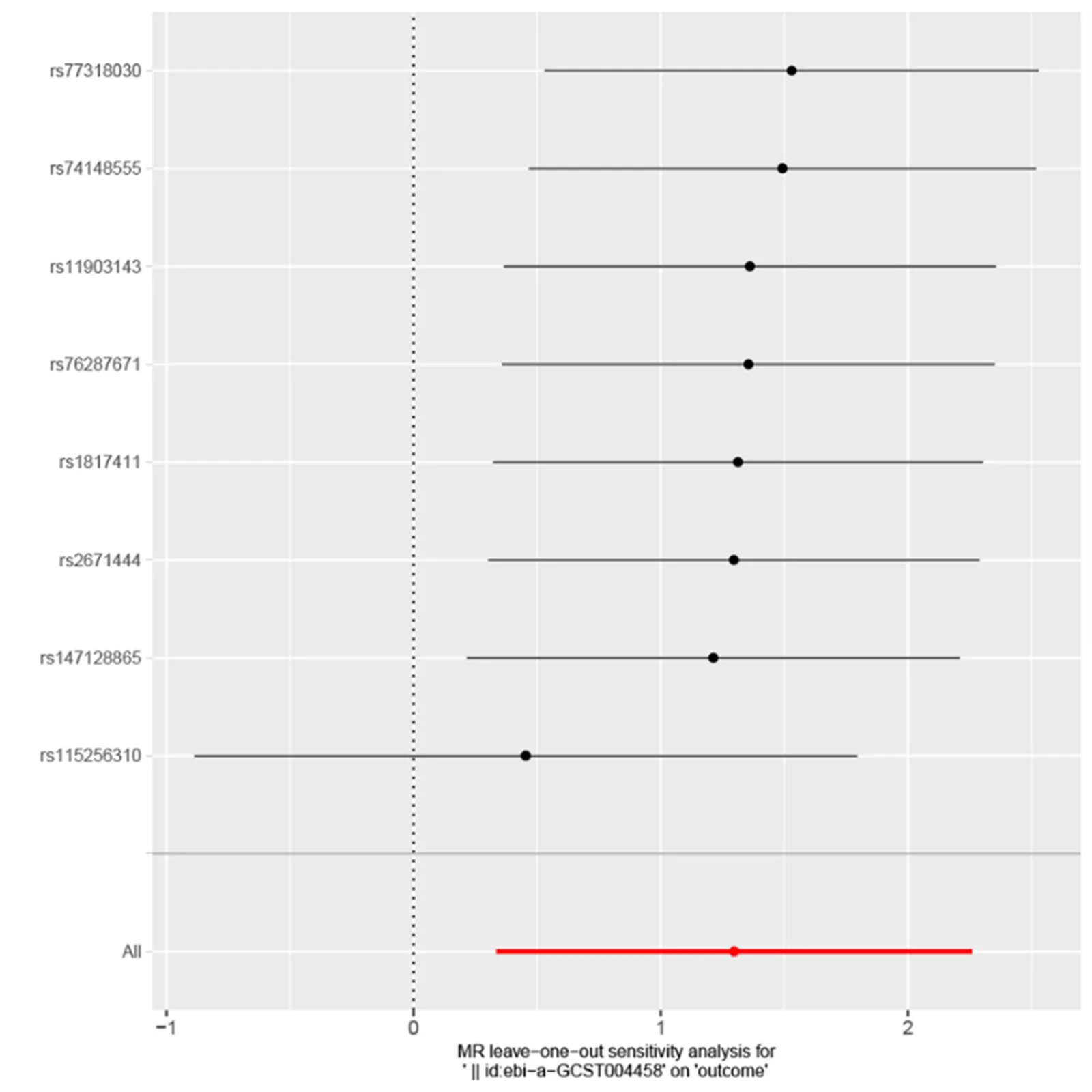
MR analysis of G-CSF levels and NPC based on leave-one-out method. C-GSF = granulocyte colony-stimulating factor, MR = Mendelian randomization, NPC = nasopharyngeal carcinoma.

After MR analysis of each inflammatory factor and NPC, it was found that there was a positive causal relationship between granulocyte colony-stimulating factor (G-CSF) and NPC (IVW-*OR*: 3.659, 95% *CI*: 1.398–9.581, *P* = .008). The results of the IVW, MR-Egger, Weighted Median, and Weighted Mode provide substantial evidence that supports the conclusion that G-CSF levels are associated with an increased risk of NPC (Table 2 and Fig. 2). The results of the simple mode analysis did not attain statistical significance; however, the OR value corroborated the hypothesis that G-CSF levels have the potential to elevate the risk of NPC (Fig. 3).

**Table 2:** MR analysis results of G-CSF levels and NPC.

MR analysis method	β	SE	OR	95% CI	*P*
MR-Egger	1.816	0.726	6.145	1.481–25.498	.046
Weighted median	1.384	0.697	3.992	1.018–15.659	.047
Inverse-variance weighted	1.297	0.491	3.659	1.398–9.581	.008
Simple mode	0.706	1.233	2.026	0.181–22.703	.585
Weighted mode	2.118	0.805	8.313	1.716–40.275	.034

95% CI = confidence interval, β = beta value, C-GSF = granulocyte colony-stimulating factor, MR = Mendelian randomization, NPC = nasopharyngeal carcinoma, OR = odds ratio*, P* = *P* value, SE = standard error.

### 3.3. The reverse MR analysis and MR Steiger test

In the reverse MR analysis, although the SNP screening criteria we applied were the same as those in forwards MR analysis, there were no suitable SNPs. This finding indicates that genetic vulnerability to NPC does not have a substantial impact on genetic predisposition to inflammatory factors. The causal relationship between NPC and inflammatory factors is influenced by complex confounding factors, necessitating discussion of the potential association between inflammatory factors and NPC.

The results of the Steiger direction test demonstrated a strong relation between SNPs and G-CSF levels (exposure factor *r*^*2*^ = 0.032 > outcome factor *r*^*2*^ = 0). The direction of the overall SNPs and each SNP affecting NPC were TRUE (*P* < .05), indicating that there is no reverse causality.

### 3.4. Sensitivity analysis

**Figure 4.**
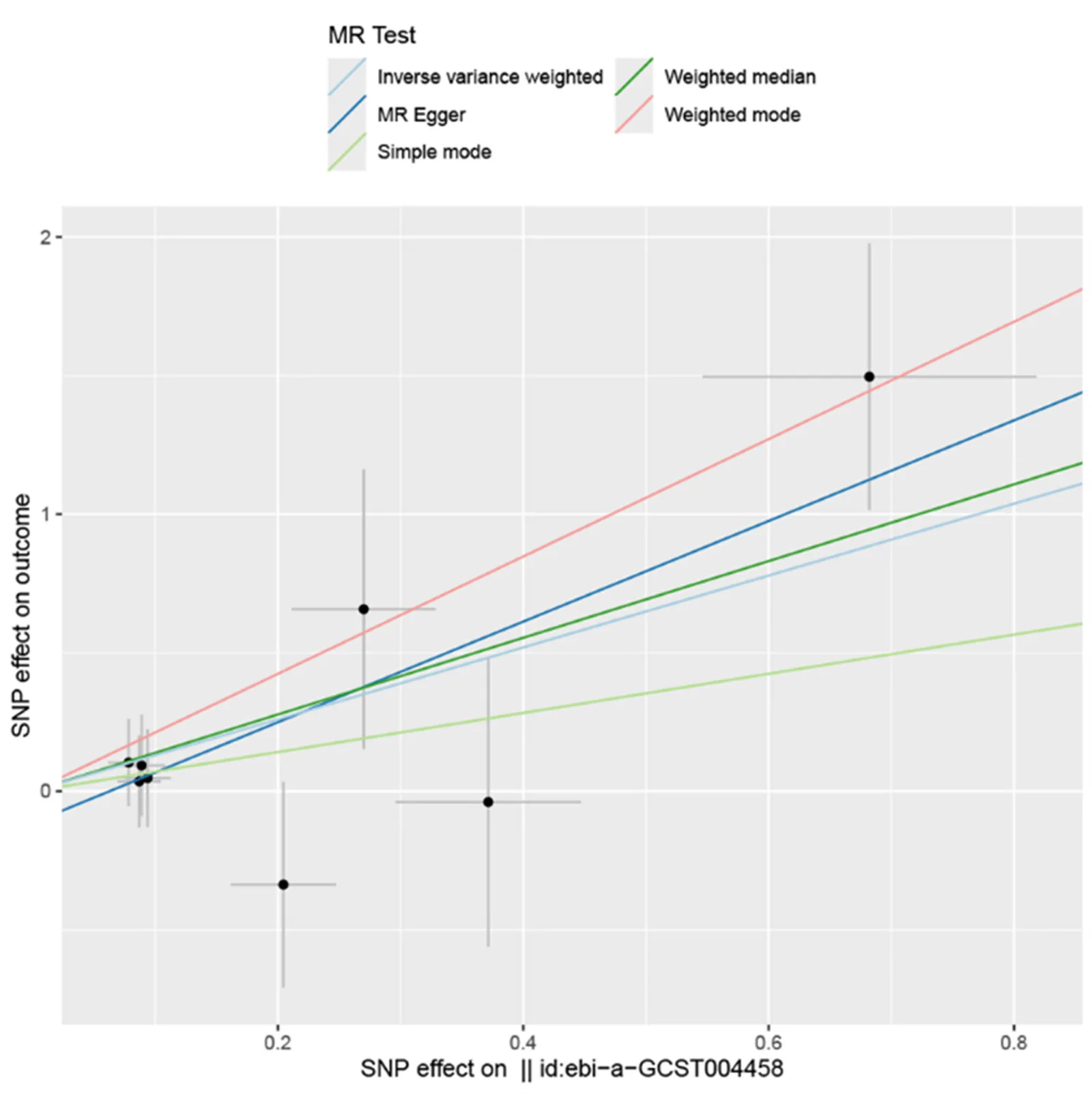
Scatter plot of MR analysis of G-CSF levels and NPC. C-GSF = granulocyte colony-stimulating factor, MR = Mendelian randomization, NPC = nasopharyngeal carcinoma.

The scatter plot demonstrated that the direction of causality was consistent across the 5 analysis methods (Fig. 4).

The Cochran *Q* test values for IVW (*P* = .537) and MR-Egger (*P* = .533) were not statistically significant, indicating that there was no heterogeneity among the included SNPs and no potential bias in the association between G-CSF levels and NPC. The value of the MR-Egger regression analysis (*P* = .370) was not statistically significant, and the intercept of −0.1142308 was close to 0, indicating that there was no horizontal pleiotropy and that the association between G-CSF levels and NPC is not influenced by other factors. The leave-one-out analysis demonstrates that the results following the exclusion of each SNP are not significantly different from the overall results, thus indicating that the results of the MR analysis are robust and not driven by a single IV.

## 4. Discussion

The inflammatory environment is a critical factor in tumor development and can influence the microenvironment of tumor growth by altering tissue homeostasis. Inflammatory factors in the TME are critical for the regulation of various cellular processes including cell proliferation, differentiation, migration, and apoptosis. Inflammatory factors play pivotal roles in the regulation of both inflammatory response and immune response processes. Inflammatory factors exert their influence on the TME through a complex network of bidirectional regulatory mechanisms. On the one hand, some inflammatory factors can stimulate the immune response to play an antitumor effect, and on the other hand, some inflammatory factors can induce the immune escape to play a tumor-promoting role.^[15]^ In consideration of the paradoxical state of inflammatory factors in tumors, the present study elected to utilize a MR analysis, with the objective of elucidating the role of inflammatory factors in the occurrence and development of NPC. According to the results of MR analysis in this study, G-CSF levels in inflammatory factors are risk factors for NPC.

G-CSF is a glycoprotein belonging to the hematopoietic growth factor, inflammatory factor, and immune stimulating factor. It is capable of promoting the differentiation and proliferation of neutrophil progenitor cells and enhancing the function of mature neutrophils. G-CSF is commonly used in the treatment of neutropenia caused by various reasons. The biological effects of G-CSF are mediated by the G-CSF receptor (G-CSFR), which is primarily involved in cell differentiation and proliferation. G-CSF has been observed to trigger the activation of signaling molecules, including pro-inflammatory transcription factors such as STAT1, STAT3, and STAT5. These molecules activate 3 major signaling pathways: PI3K/AKT, MAPK/ERK, and JAK/STAT.^[16]^ Under normal conditions, the activation of these signaling pathways leads to the maturation and mobilization of neutrophils into the bloodstream. Conversely, abnormal activation or mutation of the G-CSFR pathway can affect tumor cells directly, thereby playing a significant role in tumor growth, invasion, metastasis, angiogenesis, and immune escape.^[17]^ G-CSF and G-CSFR have been found to promote tumor progression in cellular and animal experiments,^[18–20]^ and their tumor-promoting mechanisms are as follows: enhancement of epithelial-to-mesenchymal cell transformation through M2 macrophage recruitment, which promotes tumor growth; induction of bone marrow endothelial progenitor cell mobilization, which promotes angiogenesis and tumor regeneration; activation of tumor cell gelatinases through receptors, which promotes tumor metastasis; inflammatory and immunomodulatory effects by mediating innate and adaptive immune responses, inhibiting the expression of CD4+, CD8+, and CD16 + cells, which in turn inhibits the mobilization and activity of NK cells, macrophages, and T cells, contributing to the acquisition of immune escape by tumor.^[21]^ Furthermore, elevated levels of G-CSF, along with the presence of G-CSFR expression-positive tumors, are associated with a biologically more malignant state, reduced differentiation, and a worse clinical prognosis.^[22–24]^ Relevant studies have shown that in head and neck cancer, including NPC, tumor-produced G-CSF can lead to poor patient prognosis by regulating the PI3K-AKT pathway, Mcl-1 expression, Granulocyte bone marrow-derived inhibitory cell-dependent mechanisms and caspase-3 levels. Even G-CSF has been identified as a predictor of tumor growth and poor prognosis in head and neck cancers such as nasopharyngeal carcinoma.^[25,26]^ However, given that the majority of prior studies were primarily focused on fundamental experiments and clinical trials, they were unavoidably influenced by confounding factors and reverse causation, leading to a certain degree of result bias. In the present study, MR analysis was utilized to ascertain the association between G-CSF levels and the risk of NPC at the level of genetic variation, while excluding the effect of confounding factors.

The administration of chemotherapy and radiation therapy to treat malignancies can result in bone marrow suppression, leading to a decrease in the number of neutrophils. In cases of severe neutrophil deficiency, the therapeutic or prophylactic administration of G-CSF is necessary to elevate neutrophil levels. G-CSFR is prevalent in primary and metastatic foci of head and neck tumors, and it produces G-CSF by autocrine or paracrine secretion and shows high expression.^[27,28]^ Given the direct action of exogenous G-CSF on G-CSFR-positive tumors, patients with NPC and neutrophil deficiencies are imperative to assess G-CSFR expression to select the appropriate therapeutic dose prior to G-CSF supplementation. Concurrently, G-CSF can be utilized as a potential tumor marker to observe disease progression in specific tumors or to evaluate effects of therapy. Inhibition of G-CSF and blockade of G-CSFR may offer therapeutic benefits in the treatment of specific tumors. The targeting of G-CSF and G-CSFR has emerged as a promising therapeutic strategy.^[29–31]^

## 5. Conclusion

In this study, the causal relationship and mechanism of action between G-CSF, classified as an inflammatory factor, and NPC were investigated. These findings corroborated the conclusions of previous studies that G-CSF has a causal association with the progression of NPC.

However, the study’s analysis of the causal relationship between 41 inflammatory factors and NPC in terms of MR is limited by the absence of corroborating research evidence and the need for high-quality clinical randomized controlled trials to validate the findings. The findings are subject to certain limitations; therefore, in future studies, the inclusion of data from larger samples and more rates will be necessary to draw more generalizable results. Furthermore, although MR indicates that there is a causal relationship between G-CSF and NPC, given that the study does not provide insights into the underlying mechanism, future experimental studies are still needed to explore the biological mechanism.

## Acknowledgments

The authors are grateful to the GWAS and Finngen studies.

## Author contributions

**Conceptualization:** Keyun Lin, Jiaxiang Hu, Guanying Qiao, Mei Jiang.

**Data curation:** Keyun Lin, Jiaxiang Hu, Guanying Qiao, Mei Jiang.

**Formal analysis:** Keyun Lin, Jiaxiang Hu.

**Funding acquisition:** Mei Jiang.

**Investigation:** Keyun Lin.

**Methodology:** Keyun Lin, Guanying Qiao, Mei Jiang.

**Project administration:** Keyun Lin, Guanying Qiao, Mei Jiang.

**Resources:** Keyun Lin, Jiaxiang Hu, Mei Jiang.

**Software:** Keyun Lin, Jiaxiang Hu.

**Supervision:** Keyun Lin.

**Validation:** Keyun Lin, Jiaxiang Hu.

**Visualization:** Keyun Lin, Jiaxiang Hu.

**Writing – original draft:** Keyun Lin, Jiaxiang Hu, Guanying Qiao, Mei Jiang.

**Writing – review & editing:** Keyun Lin, Jiaxiang Hu, Guanying Qiao, Mei Jiang.

## Supplementary Material
